# Outcomes of Percutaneous Fixation in Intra-articular Calcaneal Fractures

**DOI:** 10.7759/cureus.68428

**Published:** 2024-09-02

**Authors:** Jessica Thor, Raj Socklingam, Charles Kon

**Affiliations:** 1 Orthopaedic Surgery, Changi General Hospital, Singapore, SGP

**Keywords:** surgery, minimally invasive, fractures, percutaneous fixation, calcaneum

## Abstract

Displaced intra-articular calcaneal fractures have been proven to be challenging for orthopaedic surgeons worldwide due to the poor clinical outcomes. Historically, the decision whether for fixation or conservative management depended mostly on the literature of the time, initially favouring conservative management but attitudes slowly shifted to operative intervention. Percutaneous fixation options have been increasingly popular for their ability for fracture reduction without skin and wound complications of the open method. A retrospective study of 17 patients with a total of 18 calcaneal fractures treated in our hospital by a single surgeon from January 2017 to December 2019 was conducted. Fixation was done percutaneously using cannulated screws, with the patients in a lateral position. Intraoperative imaging was done using a mini-image intensifier to visualise fracture reduction, and the O-arm was used in most cases. Using the Sanders classification, there were a total of 4 IIA, 3 IIB, 2 IIC, 2 IIIAB, and 7 IIIAC. Results showed that 16 calcaneal fractures (94%) had good to excellent outcomes with the American Orthopedic Foot and Ankle Society (AOFAS) score and Maryland Foot Score (MFS), while 14 calcaneal fractures (78%) showed good to excellent outcomes with the Kerr calcaneal score. There were no wound complications encountered in our series. However, there was one patient with a k-wire broken intraoperative and left in situ and there were two patients with prominent screws. Despite this, our experience with percutaneous fixation of displaced intra-articular calcaneal fractures has been generally favourable, allowing for good outcomes and satisfactory reduction of the fracture fragments.

## Introduction

The calcaneum is the most fractured bone in the hindfoot, with an incidence of 11.5 per 100000 people per year and oft-quoted to be 2% of all fractures. It is most often seen in the context of high-energy trauma, such as a fall from height, with a gender ratio of male to female being 2.4:1 [1]. 7.21% of calcaneal fractures have been found to be associated with vertebral fractures, especially in the lumbar spine [2], with 75% of calcaneal fractures being intra-articular involving the posterior facet. Historically, calcaneal fractures were managed conservatively with non-weight-bearing and immobilisation in a cast [3] for a period of up to 6 or 8 weeks. However, non-operative management failed to restore the anatomy of the calcaneum, with inadequate articular reduction. This led to the development of better surgical approaches for open reduction and internal fixation (ORIF) of calcaneal fractures, which is now considered the gold standard for management [4] as it proved superior to conservative management with regard to footwear and return to work [5]. Other well-known complications of these fractures include malunion, development of post-traumatic subtalar joint osteoarthritis, chronic foot pain, peroneal tendinitis and lateral impingement syndrome [6]. Displaced intra-articular calcaneal fractures have been a topic of controversy in recent literature, in view of the multiple methods of surgical fixation available, ranging from ORIF to minimally invasive treatment, not to mention arthroscopic assisted means. Previously surgeons encountered many problems with the ORIF method such as infection quoted as high as 13.6% in a 2014 review by Yu et al. [7], wound and other soft tissue complications [8]. Also, local soft tissue swelling needed time to improve, further delaying surgery for days or even a few weeks [9]. This gave rise to the necessity for the development of other less invasive surgical options.

Increasingly, minimally invasive surgery (MIS) options have been explored with interest leading to more literature being published in these areas. The aim of this descriptive study is to establish the role of percutaneous fixation in calcaneal fracture fixation with the use of intraoperative imaging, achieving good patient outcomes.

## Materials and methods

**Figure 1:**
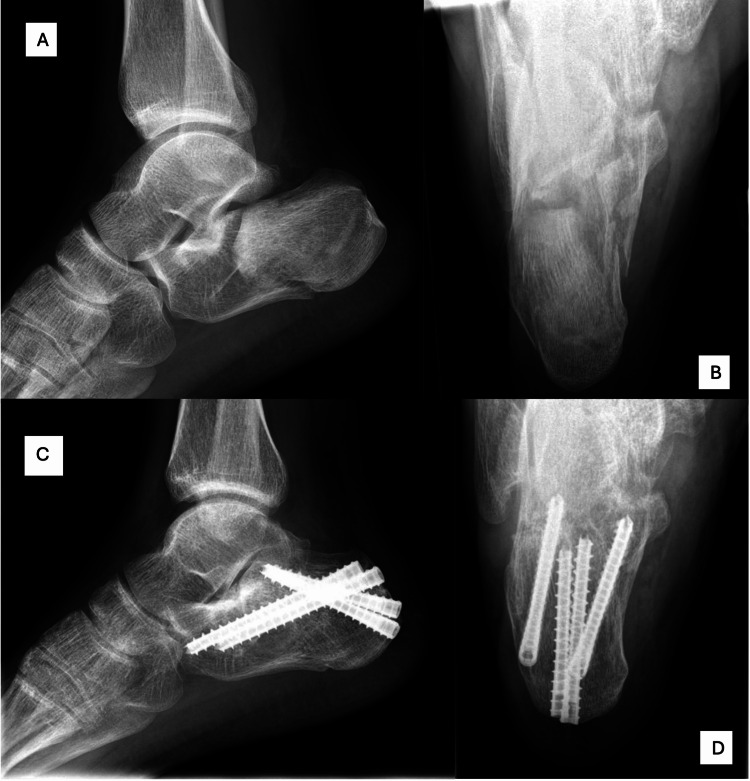
Preoperative and postoperative radiographs of the calcaneum. (A-B) Preoperative radiographs of the calcaneum and (C-D) postoperative radiographs of the calcaneum following percutaneous fixation

This is a retrospective study of patients with calcaneal fractures treated in our hospital by a single, fellowship-trained surgeon from January 2017 to December 2019. Electronic medical records and imaging of the patients were reviewed, collecting demographic data such as age and gender, as well as other information such as the mechanism of injury, past medical history, site of injury and initial Sanders classification on computed tomography (CT) scans. Every patient had CT scans done both pre-operatively and intraoperatively. The calcaneal fractures were all fixed percutaneously with cannulated screws, as shown in Figure 1, with the patient positioned in a lateral position. Intraoperatively, a mini-image intensifier was used to visualise the fracture reduction, with the addition of O-arm imaging used to ensure reduction and position of implants were satisfactory. In the immediate postoperative period, they were kept on non-weight-bearing status. All the patients were followed up with subsequent clinical review and radiographs postoperatively, with a minimum follow-up duration of at least four months. Gradually, they were allowed to progressively weight bear after clinical and radiological review at least six weeks after surgery.

Patients were then asked to complete the Maryland Foot Score (MFS), American Orthopedic Foot and Ankle Society hindfoot (AOFAS) questionnaires and Kerr calcaneal outcome score [10] after surgery. The range of motion of the ankle and the subtalar joints were also recorded as a constituent of the various outcome scores mentioned above.

Inclusion and exclusion criteria

Patients with displaced intra-articular calcaneal fractures who underwent minimally invasive fixation techniques for their calcaneal fractures in our hospital were included in the study.

Patients below the age of 18 years, pregnant women and old or conservatively managed calcaneal fractures were excluded from this study. Patients who did not undergo intraoperative computed tomography scans or meet the minimum follow-up duration of four months were also excluded from this study.

## Results

A total of 32 patients were initially identified for inclusion. However, among them, there were only 18 calcaneal fractures (17 patients) with complete data and results. One patient was excluded because he eventually underwent surgery in another hospital, and the remaining 14 patients were lost to follow-up. They were mostly foreign labourers and repatriated to their homeland following the conclusion of treatment, after about one year duration in total. Hence, 18 calcaneal fractures and 17 patients were included in this present study. They are predominantly male, with 15 men and two women. Sixteen patients suffered a unilateral injury (9 right and 7 left) while one patient suffered a bilateral injury, with a total of 18 calcaneal fractures. The mean duration of follow-up is 16 months, with a range from 4 to 33 months of follow-up. 

The most common mechanism of injury in our study population is a fall from height, with 15 (88.2%) patients, followed by road traffic accidents with two (11.8%) patients. Four (22.2%) patients in our study were smokers and one of them (5.5%) was a diabetic. There were no patients who were undergoing addiction treatment and none of the patients had metabolic disorders. This data is presented in Table 1.

**Table 1:** Demographics of patients included in the present study

Characteristics	Value (range)
Patients	17
Calcaneal fractures	18
Age in years, at time of injury	52 (39 – 70)
Smokers	4
Diabetics	1
Long-term steroid use	0
Duration of follow-up in months	16 (4 – 33)

Radiologic outcomes

**Figure 2:**
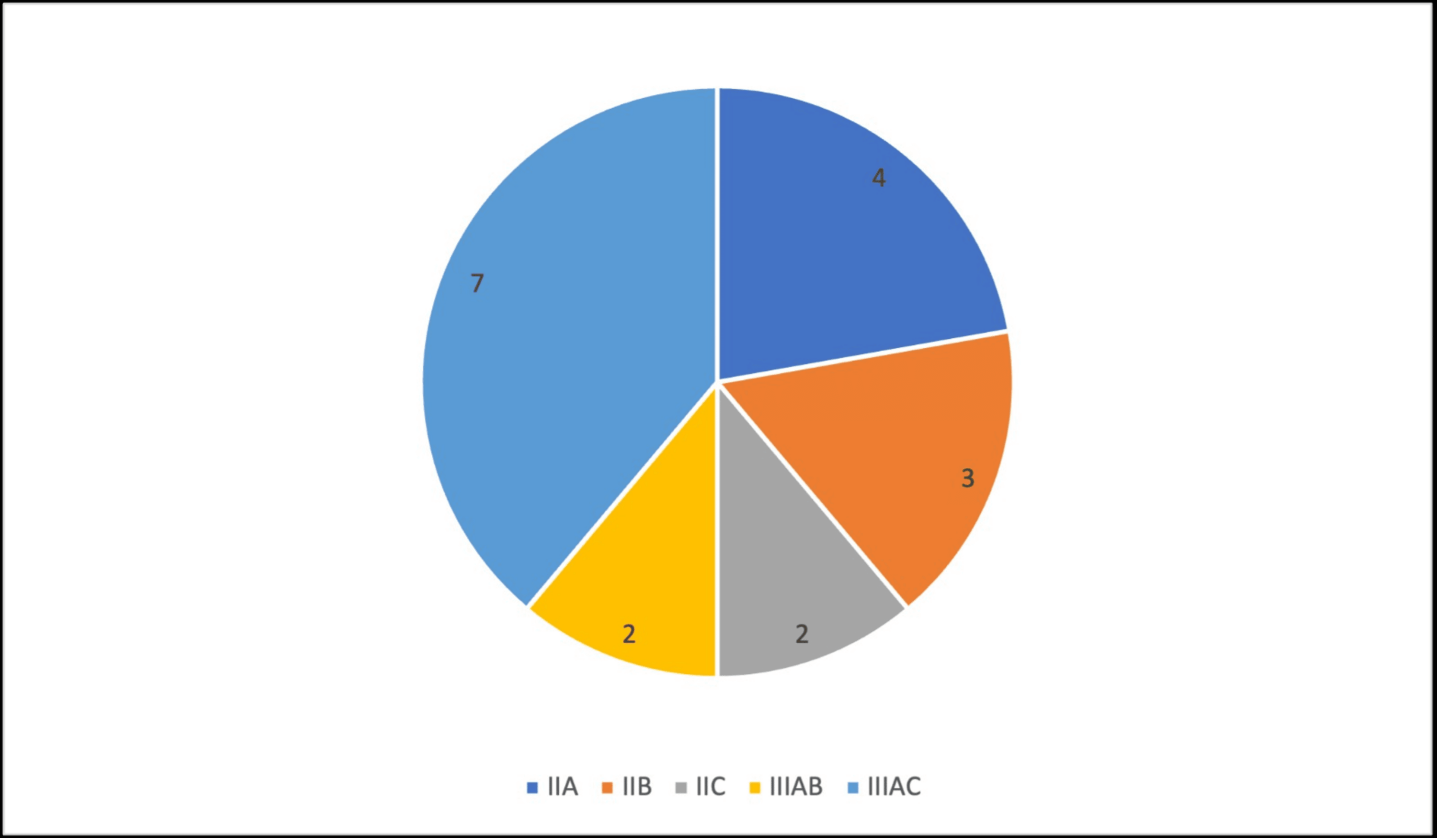
Proportion of fractures according to Sanders classification

Sanders classification [11], originally described in 1996 for intra-articular calcaneal fractures, was developed to prognosticate and guide treatment with the use of CT imaging to characterise the different fracture patterns. In our patient population, it was found that there were 38.9% IIIAC, 11.1% IIIAB, 22.2% IIA, 16.7% IIB and 11.1% IIC fractures, as presented in Figure 2.

Complications

With the use of our percutaneous method for calcaneal fixation, there were no wound complications seen. However, there was one case of a broken k-wire intraoperatively which was left in situ as it was unable to be retrieved. There was also one patient with prominent screws, requiring removal of implants after the fracture healed as the patient had persistent pain on ambulation.

Clinical outcomes

The AOFAS and MFS scores are the two most widely known and used tools to assess clinical outcomes in patients with intra-articular calcaneal fractures [12]. The Kerr outcome score proposed in 1996 [10] is a much simpler score with only four components, mainly, pain, work, walking, and walking aids developed for the specific use in calcaneal fractures.

In this study, 16 calcaneal fractures (94%) showed good (75-89) to excellent (90-100) AOFAS scores, 1 (6%) fair (50-74) and 0 (0%) poor (<50). The MFS score also showed similar outcomes, with 10 (56%) excellent (90-100), 7 (39%) good (75-89), 1 (6%) fair (50-74) and 0 (0%) poor (<50). The Kerr outcomes scores also showed 9 (50%) excellent (90-100), 5 (28%) good (75-89), 4 (22%) fair (50-74) and 0 (0%) poor (<50). These scores are presented in Table 2.

**Table 2:** Outcome scores post percutaneous calcaneal fixation

Outcome scores	Excellent	Good	Fair	Poor	Mean (range)
AOFAS (American Orthopedic Foot and Ankle Society) hindfoot scores	9	8	1	-	86 (59 – 100)
MFS (Maryland Foot Score)	10	7	1	-	90 (69 – 100)
Kerr	9	5	4	-	83 (50 – 100)

## Discussion

Displaced intra-articular calcaneal fractures have always been difficult to manage and obtain good outcomes for patients. The main outcomes to achieve are painless, functional and shoe-able feet in these patients to allow for a good quality of life following injury to the calcaneum. This can be achieved via restoring subtalar articular congruity and calcaneal shape, including the width, height and alignment, and minimising complications.

In the past, calcaneal fractures were treated conservatively as surgical intervention had no proven superiority. However, recent literature has shown some benefits of surgical fixation in displaced intra-articular calcaneal fractures. Furthermore, the use of antibiotics, better AO principles of fixation, computed tomography and fluoroscopy aided surgeons greatly in operative management [13]. Not to mention, subtalar joint incongruity of as little as 1 to 2mm can lead to drastic alterations in the weight bearing mechanics of the foot which may in turn result in an earlier onset of post-traumatic arthritis. Hence, conservative management may not be as favourable as before. Basile in 2010 was able to show that operative treatment achieved a better AOFAS score in contrast to non-operative management [14]. Agren et al. also produced promising results in the long run of about 8 to 12 years, with a better physical component of Short Form-36 (SF-36), pain and function visual analogue scale in the operative group, showing a higher prevalence of radiographic post-traumatic subtalar arthritis in the non-operative group simultaneously [9]. A systematic review by Veltman et al. showed a mean AOFAS score of 73.7 with the ORIF method of surgical fixation [15]. Despite the dearth of quality evidence promoting surgical fixation of calcaneal fractures over non-operative management, others have also found variable degrees of success in less invasive surgical methods. However, Law et al. were able to achieve an excellent mean AOFAS score of 90.3 at 2 years using combined methods of subtalar arthroscopy associated percutaneous fixation [16]. Abdelgaid was also able to achieve a mean AOFAS score of 89.26 with 79.23% of patients achieving good to excellent scores using closed reduction and percutaneous fixation methods [17]. Similarly, our study showed a mean AOFAS of 86, with 94% good to excellent results following percutaneous fixation.

MIS fixation techniques have been praised for their numerous benefits such as faster recovery and wound healing with smaller incisions that do not compromise the soft tissue envelope. This can be especially helpful in patients with relative contraindications to ORIF, such as heavy smokers, poorly controlled diabetics or vascular compromised. Stulik et al. reported 20 superficial infections and 5 deep infections in percutaneous fixation of the calcaneum which amounted to infection rates of 8.71% [18]. This is remarkable compared to the infection and skin flap necrosis rates of 13.6% with the ORIF method found in the review by Yu et al. [7]. In our series, no wound complications were found.

Currently, most critics may feel that MIS may not be suitable for all types of calcaneal fractures and that it is best suited to tackle simpler fracture patterns [19] such as a two-part fracture [20]. Our experience with percutaneous fixation methods in Sanders II and III type fractures yielded favourable results and is consistent with analysis by Wallin et al. [21] that percutaneous fixation can be considered for Sander’s type II, III and IV calcaneal fractures.

Worker’s compensation was also found to be an important factor when considering the results of calcaneal fracture treatment. According to Buckley et al., operative management was not shown to be superior to conservative management [22]. However, when patients receiving worker’s compensation were removed, operative outcomes were found to be significantly better. In our study, we were able to demonstrate good outcomes despite two (11.8%) of our patients receiving worker’s compensation.

The limitations of our study include the small cohort size as well as the lack of long-term outcomes in our patients. This can be attributed to the fact that these injuries were generally sustained by manual labourers, contributing to a significant proportion of our study population, who have since returned to their native countries following the completion of surgical treatment and are lost to follow-up. Hence, they were excluded from our study as outcome scores and follow-up data were not obtained.

## Conclusions

Percutaneous fixation of displaced intra-articular calcaneal fractures is a reliable and reproducible method with good outcomes, both clinical and radiographic ones. It has shown to be versatile in the use of fixation in various patterns of calcaneal fractures.

## References

[1] Mitchell MJ, McKinley JC, Robinson CM (2009). The epidemiology of calcaneal fractures. Foot (Edinb).

[2] Walters JL, Gangopadhyay P, Malay DS (2014). Association of calcaneal and spinal fractures. J Foot Ankle Surg.

[3] Eastwood WJ (1938). Fracture of the os calcis. British J Surg.

[4] Guerado E, Bertrand ML, Cano JR (2012). Management of calcaneal fractures: what have we learnt over the years?. Injury.

[5] Gougoulias N, Khanna A, McBride DJ, Maffulli N (2009). Management of calcaneal fractures: systematic review of randomized trials. Br Med Bull.

[6] Pozo JL, Kirwan EO, Jackson AM (1984). The long-term results of conservative management of severely displaced fractures of the calcaneus. J Bone Joint Surg Br.

[7] Yu X, Pang QJ, Chen L, Yang CC, Chen XJ (2014). Postoperative complications after closed calcaneus fracture treated by open reduction and internal fixation: a review. J Int Med Res.

[8] Shuler FD, Conti SF, Gruen GS, Abidi NA (2001). Wound-healing risk factors after open reduction and internal fixation of calcaneal fractures: Does correction of Bohler’s angle alter outcomes?. Orthoped Clin North Am.

[9] Agren PH, Wretenberg P, Sayed-Noor AS (2013). Operative versus nonoperative treatment of displaced intra-articular calcaneal fractures: a prospective, randomized, controlled multicenter trial. J Bone Joint Surg Am.

[10] Kerr PS, Prothero DL, Atkins RM (1996). Assessing outcome following calcaneal fracture: a rational scoring system. Injury.

[11] Sanders R (1992). Intra-articular fractures of the calcaneus: present state of the art. J Orthop Trauma.

[12] Schepers T, Heetveld MJ, Mulder PG, Patka P (2008). Clinical outcome scoring of intra-articular calcaneal fractures. J Foot Ankle Surg.

[13] Sanders R (2000). Displaced intra-articular fractures of the calcaneus. J Bone Joint Surg Am.

[14] Basile A (2010). Operative versus nonoperative treatment of displaced intra-articular calcaneal fractures in elderly patients. J Foot Ankle Surg.

[15] Veltman ES, Doornberg JN, Stufkens SA, Luitse JS, van den Bekerom MP (2013). Long-term outcomes of 1,730 calcaneal fractures: systematic review of the literature. J Foot Ankle Surg.

[16] Law GW, Yeo NE, Yeo W, Koo K, Chong KW (2017). Subtalar arthroscopy and fluoroscopy in percutaneous fixation of intra-articular calcaneal fractures. J Orthop Surg (Hong Kong).

[17] Abdelgaid SM (2012). Closed reduction and percutaneous cannulated screws fixation of displaced intra-articular calcaneus fractures. Foot Ankle Surg.

[18] Stulik J, Stehlik J, Rysavy M, Wozniak A (2006). Minimally-invasive treatment of intra-articular fractures of the calcaneum. J Bone Joint Surg Br.

[19] Swanson SA, Clare MP, Sanders RW (2008). Management of intra-articular fractures of the calcaneus. Foot Ankle Clin.

[20] Coetzee JC, Pena FA (2016). Minimally invasive closed reduction and internal fixation of calcaneal fractures. Minimally Invasive Surgery in Orthopedics.

[21] Wallin KJ, Cozzetto D, Russell L, Hallare DA, Lee DK (2014). Evidence-based rationale for percutaneous fixation technique of displaced intra-articular calcaneal fractures: a systematic review of clinical outcomes. J Foot Ankle Surg.

[22] Buckley R, Tough S, McCormack R, Pate G, Leighton R, Petrie D, Galpin R (2002). Operative compared with nonoperative treatment of displaced intra-articular calcaneal fractures: a prospective, randomized, controlled multicenter trial. J Bone Joint Surg Am.

